# L-Tryptophan Stimulates Bioactive Metabolite Accumulation and Cell Wall Remodelling in Flax Callus Cultures

**DOI:** 10.3390/molecules31081229

**Published:** 2026-04-08

**Authors:** Kornelia L. Tudruj, Michał Piegza, Lucyna Dymińska, Maja Słupczyńska, Magdalena Wróbel-Kwiatkowska

**Affiliations:** 1 Department of Biotechnology and Food Microbiology, Faculty of Biotechnology and Food Science, Wroclaw University of Environmental and Life Sciences, 51-630 Wrocław, Poland; 122519@student.upwr.edu.pl (K.L.T.); michal.piegza@upwr.edu.pl (M.P.); 2 Department of Bioorganic Chemistry, Faculty of Production Engineering, Wroclaw University of Economics and Business, 53-345 Wrocław, Poland; lucyna.dyminska@ue.wroc.pl; 3 Department of Animal Nutrition and Feed Science, Wrocław University of Environmental and Life Sciences, 51-630 Wrocław, Poland; maja.slupczynska@upwr.edu.pl

**Keywords:** *Linum usitatissimum* L., L-tryptophan, biostimulant, fourier transform infrared spectroscopy (FTIR), flavonoids, phenolic compounds

## Abstract

While L-tryptophan is a precursor of plant growth regulators, its effects on secondary metabolism, amino acid profile and cell wall organization in flax callus remain underexplored. This study aimed to optimize flax callus shaken cultures and evaluate the impact of L-tryptophan (0.1 mM and 1 mM) on structural properties of plant cell walls in tested callus using Fourier transform infrared spectroscopy. The impact of L-tryptophan on callus proliferation and metabolism was also determined, because amino acids (among them L-tryptophan) can promote the growth of callus. The results showed that 1 mM L-tryptophan is an effective elicitor, which stimulates flax callus to accumulate larger amounts of bioactive compounds, especially carotenoids and polyphenols, than control callus cultured without L-tryptophan. A lower concentration of L-tryptophan (0.1 mM) slightly improved the level of determined secondary metabolites (except flavonoids). The effect of L-tryptophan on polymers in plant cell walls was investigated. The data confirm that the plant cell wall is a dynamic structure, capable of remodelling in response to growth conditions and external agents. L-tryptophan (0.1 and 1 mM) reduced cellulose levels and induced structural changes in cellulose compared to the untreated control. The structural analyses also suggested a decrease in lignin level and increase in pectin amounts in flax callus after tryptophan addition in comparison to control callus. The results may reflect the relationship between tryptophan and auxins (which are derived from tryptophan) and confirm the role of these metabolites in shaping the structure of the plant cell wall. In fact, an increase in tryptophan level was confirmed in flax callus in tested experimental conditions (supplementation of cultures with both doses of L-tryptophan). These findings have practical significance, because L-tryptophan is also used as a fertilizer or component of fertilizers in plant cultivation.

## 1. Introduction

Flax (*Linum usitatissimum* L.) has been cultivated since ancient times due to its wide-ranging applications: its seeds are applied by the food and pharmaceutical industries, while its fibres are used by the textile and automotive industries. Flax is biochemically rich; its seeds (linseed) are a source of lignans and omega-3 fatty acids, which have a beneficial effect on hormonal balance and prevent cardiovascular diseases [1]. Flax fibres, in contrast to cotton fibres, contain not only cellulose but also hemicellulose, pectin and lignin. These compounds, especially lignin and its constituents, exhibit potent antioxidant properties [2]. Lignin has been reported to possess antimicrobial, antifungal and antioxidant properties [3,4], which are crucial for plant protection against pathogens and stress conditions (e.g., UV radiation) [5]. On the other hand, these compounds may be extracted from the plant tissues or used—as constituents of fibres or seeds—for biomedical applications. Another polymer present in flax fibre is hemicellulose, which exhibits bioactive properties and shows potential for applications in drug delivery and wound-healing materials [6]. The main constituent of flax fibre is cellulose, which is a non-toxic, biodegradable, recyclable and natural polymer with high chemical reactivity [7], supporting application of flax fibres for preparation of biocomposites with medical potential [8]. Notably, these compounds—cellulose, hemicellulose, lignin, and pectin—are the primary polymers forming the cell wall of all plant cells.

The plant material used in the present study was flax of the fibre cultivar Nike. Sterilized seeds of this cultivar were germinated in aseptic tissue cultures, and the obtained seedlings were applied for callus initiation. In nature, callus is a tissue induced in response to wounding [9]. In laboratory experiments, callus is used to generate genetically modified and horticultural plants [10] and serves as a versatile system in shaken cultures for synthesis of bioactive compounds, called phytochemicals. These compounds exhibit antimicrobial, anticancer, anti-inflammation, wound-healing and antioxidant properties [11,12,13] or can be classified as recombinant proteins: antibodies [14] and vaccines [15,16]. Callus cultures can be maintained on agar-solidified medium or as suspension cultures, with shaken callus cultures functioning as bioreactors [17].

Thus, callus cultures have high biotechnological potential in pharmacy and agriculture, and they can be used for the synthesis of many therapeutic compounds such as taxol, vincristine, vinblastine and metabolites with antioxidant properties (vitamin C, polyphenols, carotenoids, etc.) [10,18].

The aim of this study was to optimize shaken callus cultures of flax to increase the levels of secondary bioactive compounds (polyphenols, flavonoids) by supplementing the cultures with L-tryptophan. This aromatic amino acid is involved in many pathways in plant metabolism, leading to the synthesis of phytohormones (e.g., auxins), which influence polyphenol synthesis and impact phytoalexins (e.g., camalexin), which exhibit antimicrobial, antifungal and anticancer potential [19,20]. Moreover, metabolites derived from L-tryptophan play a role in plant responses to stresses (both biotic and abiotic) by activating plant defence mechanisms called systemic acquired resistance (SAR) and induced systemic resistance (ISR) [19].

The obtained callus, after cultivation with L-tryptophan in two different concentrations (0.1 mM and 1 mM), was subjected to Fourier transform infrared spectroscopy (FTIR) testing to evaluate the impact of this amino acid on plant cell wall composition and organization. FTIR is a powerful analytical method, which can be applied in medicine, criminalistics and food analysis to observe potential changes, for example, in food during processing or for adulteration of food products [21]. In the case of medicine, this technique can be applied to monitor various compounds, including hormones, glucose in blood samples or in discriminant analysis. In this study, a semi-quantitative FTIR method [22] was used to evaluate cellulose, hemicellulose and lignin in flax callus after supplementation with L-tryptophan in two tested concentrations, 0.1 mM and 1 mM.

## 2. Results and Discussion

### 2.1. Impact of Tryptophan Supplementation on Photosynthetically Active Pigments

**Figure 1:**
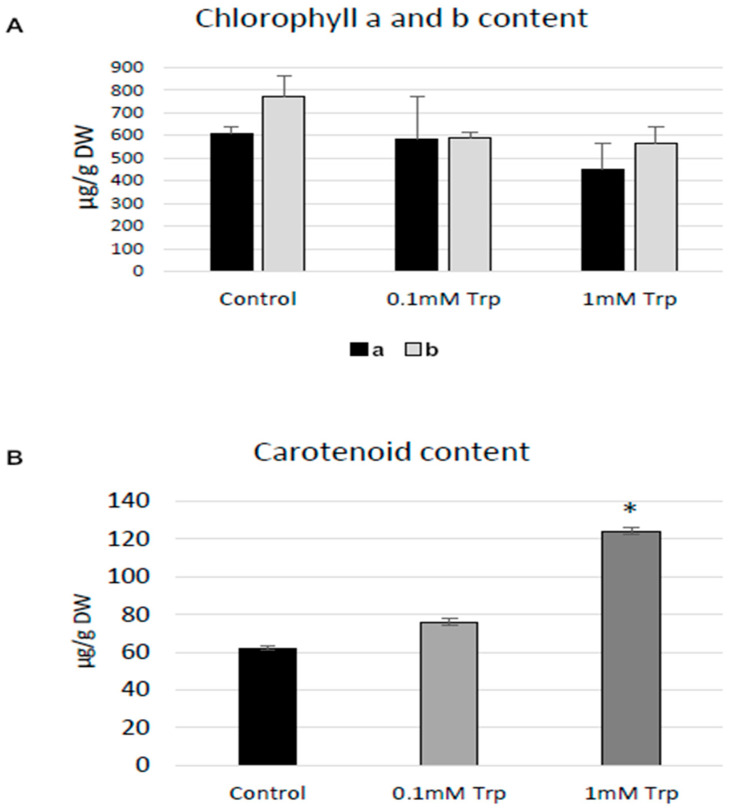
Effect of L-tryptophan on photosynthetically active pigments in callus shaken cultures. The cultures were treated with L-tryptophan in two different concentrations: 0.1 mM and 1 mM L-tryptophan. Chlorophyll a and b (**A**) and carotenoid (**B**) contents were measured as described in Section 3. Values are the means ± SD of three samples. The statistical analysis was performed using Student’s *t*-test (* *p* < 0.05).

Addition of 0.1 mM and 1 mM tryptophan to callus shaken cultures of flax (cv. Nike) increased the carotenoid content, while the level of chlorophyll a was not changed or slightly decreased in cultures treated with 1 mM Trp (Figure 1). Addition of L-tryptophan in both tested concentrations reduced the level of chlorophyll b. Contrasting findings were obtained by another study [23], which noted higher levels of chlorophyll a in maize in response to tryptophan; however, those experiments were carried out on whole plants grown in pots, and tryptophan was applied to the soil.

Thus, it can be assumed that in the present study, a positive effect of L-tryptophan on the level of carotenoids was observed, but these results were not statistically significant.

### 2.2. Effect of L-Tryptophan on Flax Callus Growth

**Figure 2:**
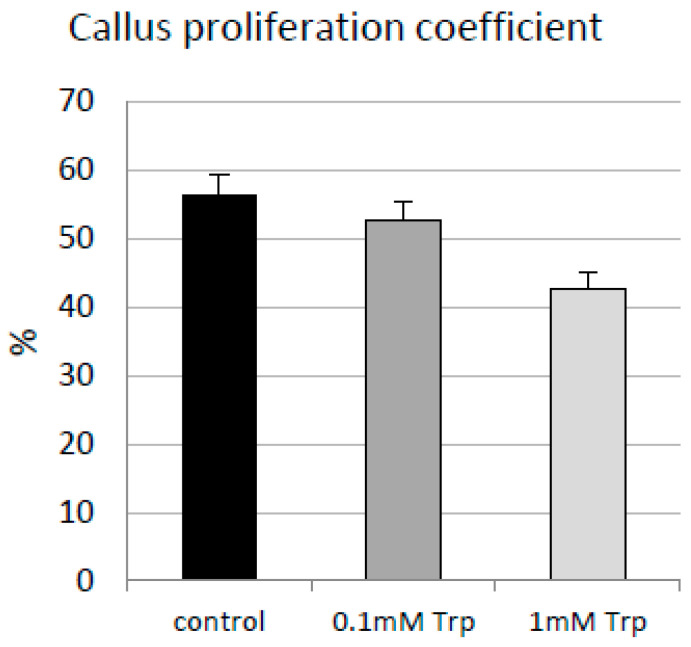
Effect of L-tryptophan on flax callus proliferation. Two different concentrations (0.1 mM and 1 mM) of L-tryptophan were tested in callus shaken cultures of the flax cultivar Nike. The parameter was calculated as described in Section 3.

L-tryptophan (L-Trp) in low concentrations may act as a stimulant for plant metabolism and growth. Therefore, callus biomass was measured in the tested cultures, and a callus proliferation coefficient was calculated. In each experiment—control and in treatment with L-Trp—an increase in final plant tissue biomass was noted in the performed cultures. However, the addition of L-Trp caused a lower biomass increase compared to the control cultures (Figure 2). The highest measured biomass was observed for the control cultures without any supplementation, for which the measured callus proliferation coefficient was equal to 56.4%. For the cultures with the addition of 0.1 mM L-Trp, a lower callus proliferation coefficient was determined (finally, about 52% growth of biomass was detected), while the supplementation of 1 mM L-Trp caused the lowest values of the callus proliferation coefficient (42%). The observed changes in callus biomass and its proliferation align with earlier studies performed on whole flax plants in tissue culture conditions, which showed that L-Trp in tested doses of 1 mM and 10 mM negatively influenced root formation [24]. It was also found that 1 mM L-Trp resulted in even lower biomass of flax plants. In this study, no reduction in biomass was observed, but slower callus proliferation was noticed after treatment with tryptophan. The primary reason may be the observed lower chlorophylls levels, especially chlorophyll b, which is a critical factor influencing plant biomass [25].

On the other hand, L-Trp in a low concentration added to the soil may positively impact plant biomass; however, in this case, L-Trp mainly influences the microorganisms in soil, promoting plant growth [26]. In the present study, sterile tissue cultures were examined, so the effect of L-Trp only on plant cells was analyzed. The effect of tryptophan on plant metabolism and physiology depends on the concentration of these amino acids. It was reported that Trp in a concentration between 0.25 mM and 5 mM applied to strawberry fruits caused different effects on phenotypic and metabolic properties of these fruits, leading to the conclusion that a Trp concentration of 2 mM produced the best results [27]. This concentration is higher than that used in the present study, but in the cited study, 2 mM Trp was applied to strawberry fruits by immersion for 5 min, while in present study, the flax callus was cultured with this amino acid for 14 days of culture in a liquid medium.

### 2.3. Determination of Secondary Metabolites in Callus Shaken Flax Cultures

**Figure 3:**
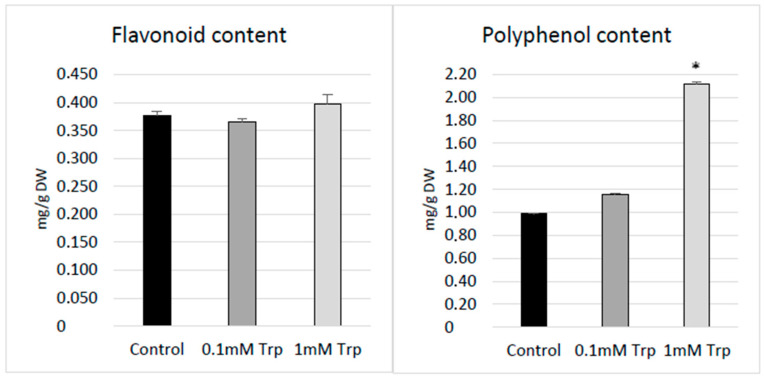
Levels of secondary metabolites (phenolic and flavonoids) measured in flax callus cultured in shaken cultures with supplementation of L-tryptophan (0.1 mM or 1 mM L-Trp); control cultures were performed without any supplementation. Values are the means ± SD of three samples. The statistical analysis was performed using Student’s *t*-test (* *p* < 0.05).

Supplementation of callus shaken cultures with 1 mM L-tryptophan resulted in increased levels of all analyzed secondary metabolites (i.e., flavonoids and polyphenols). The strongest effect was observed for polyphenols, which exhibited more than two-fold higher levels (after treatment with 1 mM L-Trp) compared to the control, untreated callus (Figure 3). The observed increase in the phenolic compound level after L-tryptophan supplementation is in accordance with the literature data noted, for example, for strawberries. In these experiments, plants treated with this amino acid showed activated phenolic biosynthesis and improved antioxidant capacity [27]. The potential mechanism for inducing phenolic synthesis by tryptophan may be indirect activation of an enzyme, phenylalanine ammonia-lyase (PAL), which is crucial for the phenylpropanoid pathway [28]. The first step of synthesis of two aromatic amino acids—tryptophan and phenylalanine (which is the precursor for many different phenolic compounds)—is the same and occurs via the shikimate pathway [29]. In this pathway, the common stage in the synthesis of both amino acids (tryptophan and phenylalanine) is chorismate synthesis. It was reported that the enzyme anthranilate synthase converts chorismate to anthranilate (which is then converted to tryptophan), and this enzyme is negatively regulated by tryptophan through feedback inhibition [29]. Thus, it can be speculated that the applied high concentration of tryptophan in callus shaken cultures could be a reason for the negative regulation of anthranilate synthase, so the chorismate was possibly directed into the phenylalanine and tyrosine synthesis pathway. Thus, manipulation of the tryptophan level may be the reason for larger amounts of phenylalanine, which is the substrate for phenylpropanoids [30].

In the case of flavonoids, a slight increase was observed, but only in the case of callus cultured with addition of 1 mM L-tryptophan, and these results were not statistically important (Figure 3). The findings may suggest changes in metabolic distribution in flax callus after tryptophan treatment. On the other hand, the weaker response of flavonoids compared to total polyphenols may result from the fact that flavonoids are mainly synthesized in plants in response to specific conditions, e.g., environmental stresses (salinity, water stress, UV radiation, etc.) and in interactions between plants and mycorrhizal fungi and pathogens [31], while other phenolic compounds have rather broad-spectrum action. It should be also pointed out that the obtained data are in accordance with the results obtained for amaranth callus, for which tryptophan did not have the positive effect on flavonoid level (Trp, dependent on concentration, even caused a reduction in flavonoid content), while the other aromatic amino acids (tyrosine and phenylalanine) had beneficial effect on flavonoid accumulation [32].

### 2.4. Amino Acid Analysis of Flax Callus Using HPLC-DAD Method

The performed analysis revealed that exposure of flax callus to L-tryptophan caused changes in the profile of amino acids (Table 1). L-tryptophan levels were highest in callus treated with 1 mM L-tryptophan and were almost 5-fold higher than in the control callus. Exposure of flax callus to lower concentrations (0.1 mM) resulted in an increase, but only by about 27% compared to the control callus cultures. These results indicate that L-tryptophan can be taken up by plant cells in culture.

Interestingly, the addition of L-tryptophan at both doses reduced the phenylalanine content in flax callus. The lowest observed phenylalanine concentration was confirmed in callus after exposure to 1 mM L-tryptophan, which may suggest that phenylalanine was redirected to phenylpropanoid synthesis, as significantly higher levels of total phenolic compounds were determined in these calli.

It can also be assumed that all applied treatments caused a negative regulation of anthranilate synthase, resulting in higher tyrosine levels (confirmed by the analysis). The measured lower proline levels in calli supplemented with 1 mM L-tryptophan may indicate that these calli were grown under optimal conditions, as proline is induced in plant cells under stress conditions. In fact, it has been found that L-tryptophan may be an osmolyte that regulates osmotic pressure within the cell [33]. The cited article also noted that foliar application of tryptophan to peanut plants resulted in increased proline levels, but in these experiments, the plants were grown in pots and subjected to drought stress, whereas in the present study, callus tissue was analyzed and stress conditions were not tested.

### 2.5. Analysis of Plant Cell Wall Polymers by FTIR

**Figure 4:**
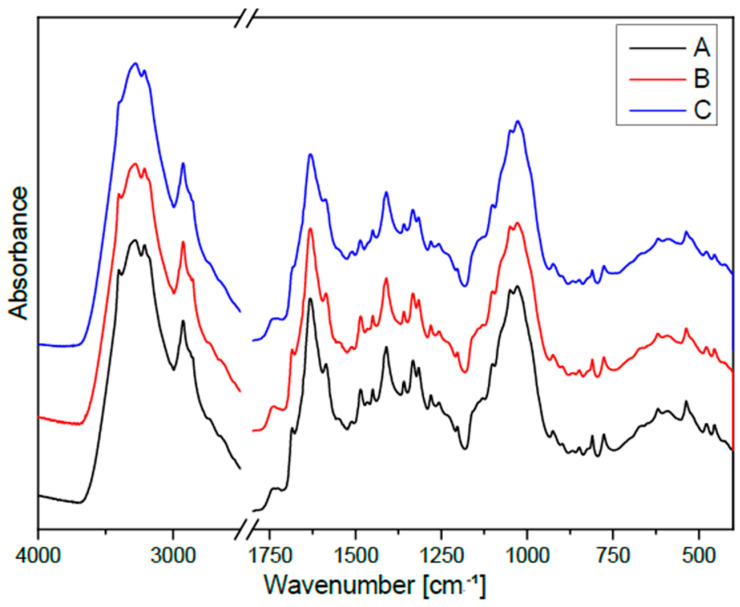
FTIR spectra of the studied samples: A—control callus obtained from flax shaken cultures (of cultivar Nike); B—callus cultured in shaken cultures with supplementation of 0.1 mM L-tryptophan; C—callus obtained from flax shaken cultures supplemented with 1 mM L-tryptophan.

**Figure 5:**
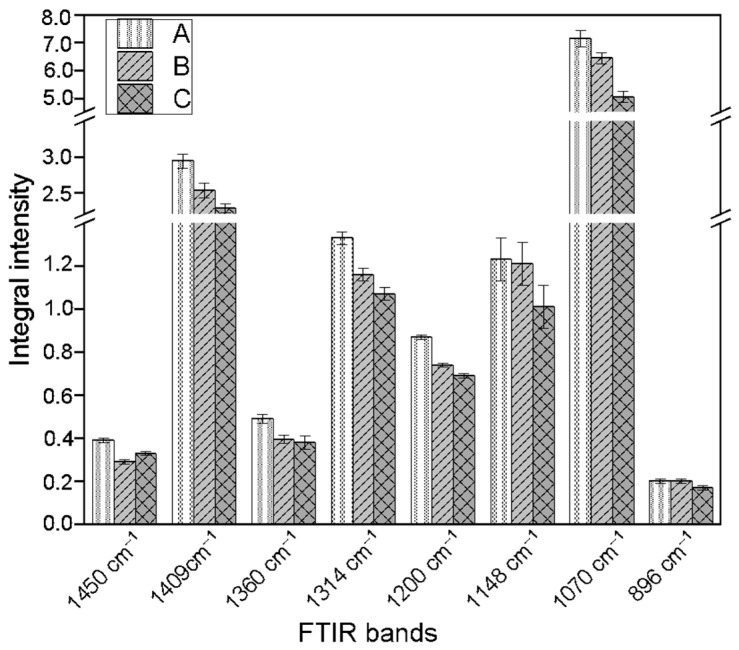
Differences in the integral intensities of FTIR absorption bands corresponding to characteristic cellulose bond vibrations across the studied samples: A—control callus, obtained from shaken cultures not treated with L-tryptophan; B—flax callus derived from cultures treated with 0.1 mM L-tryptophan; C—flax callus in cultures supplemented with 1 mM L-tryptophan.

The FTIR spectrum exhibits absorption bands characteristic of cellulose (Figure 4). The band observed at approximately 1450 cm^−1^ arises from overlapping C-H bending, CH_2_ rocking, and O-H deformation vibrations (Figure 5). The band near 1410 cm^−1^ is attributed to stretching vibrations of the glucose ring combined with bending modes of C-H and O-H groups. The absorption band at 1360 cm^−1^ originates from coupled CH_2_ bending, glucose ring stretching, and O-H deformation vibrations, while the band at around 1315 cm^−1^ is associated with C-H bending vibrations within the cellulose framework. The band near 1200 cm^−1^ is commonly associated with bending vibrations of hydroxyl groups engaged in hydrogen bonding. The band at 1148 cm^−1^ is characteristic of C-O-C stretching vibrations in glycosidic bonds linking glucose units. In addition, the absorption at approximately 1077 cm^−1^ corresponds to C-O stretching vibrations of ether and alcohol groups present in the polysaccharide backbone. The band observed near 896 cm^−1^ is associated with glucose ring vibrations and CH_2_ rocking modes.

The integral intensities of the bands characteristic of cellulose vibrations in the callus samples after addition of tryptophan (0.1 mM, as well as 1 mM) are slightly lower than in the control, untreated flax callus, which suggest a somewhat lower cellulose content compared to the control samples. The samples treated with L-Trp in both concentrations also showed reduced integral intensities of the band associated with hydrogen bond vibrations, suggesting weaker interactions between cellulose chains in cell walls of the tested samples in comparison to the control callus. The observed changes in the cellulose level and structure might be an indirect result of added L-tryptophan because this metabolite is a precursor of auxins, which cause thickening of cell walls [34]. The data also confirm the dynamic nature of the plant cell wall, which changes in response to different growth conditions and is dependent on the state of the plant cell.

### 2.6. Impact of L-Trp on Lignin Level in Flax Callus

**Figure 6:**
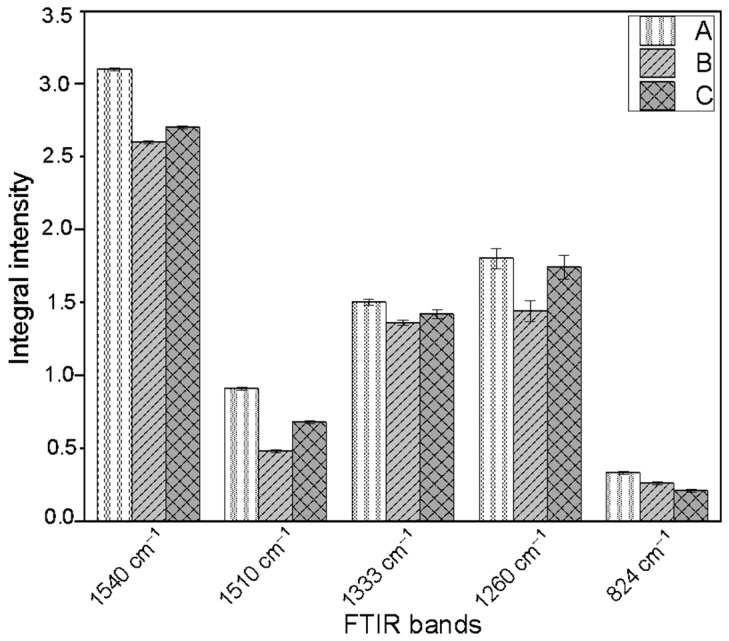
Differences in the integral intensities of FTIR absorption bands corresponding to characteristic lignin bond vibrations across the studied samples: A—control callus; B—callus derived from shaken cultures of flax supplemented with 0.1 mM L-Trp; C—flax callus derived from cultures treated with 1 mM L-Trp.

The FTIR spectrum of lignin is characterized by bands typical of aromatic and phenolic structures, which clearly confirm its presence in plant cell walls. The band observed around 1540 cm^−1^ is attributed to C=C stretching vibrations within aromatic rings, which constitute a fundamental structural element of lignin (Figure 6). Another intense band located near 1510 cm^−1^ is associated with aromatic ring vibrations and is widely used as a quantitative indicator of lignin in complex plant-derived materials. The band at 1333 cm^−1^ corresponds to a combination of C-O stretching vibrations and aromatic ring vibrations. In turn, the signal observed at approximately 1260 cm^−1^ arises from overlapping C-C and C-O stretching vibrations together with deformation vibrations of carbonyl groups, which are characteristic of guaiacyl and syringyl units, the primary building blocks of lignin polymers. The presence of a band at 824 cm^−1^, related to out-of-plane bending vibrations of aromatic C–H bonds and ring deformations, further confirms the occurrence of substituted aromatic structures.

Integral intensities of bands characteristic of lignin fulfil the following relations:

- Band at 1540 cm^−1^: Nike control > Nike with 1 mM L-Trp ≈ Nike with 0.1 mM L-Trp;
- Bands at 1510, 1333 and 1260 cm^−1^: Nike control > Nike with 1 mM L-Trp > Nike with 0.1 mM L-Trp;
- Band at 824 cm^−1^: Nike control > Nike with 0.1 mM L-Trp > Nike with 1 mM L-Trp.

The control callus is characterized by the highest integral intensity values for all analyzed bands. The samples of callus cultured with 0.1 mM L-Trp show systematically lower values, while callus supplemented with 1 mM L-Trp occupies an intermediate position. The data suggest lower lignification in the callus samples after addition of L-Trp.

Notably, levels of cellulose and lignin are related to cell wall synthesis [35], and the results reflect the impact of L-Trp on the structure and organization of the plant cell wall. This observation is especially important because L-Trp is applied as a fertilizer or component of fertilizers for plant cultivation.

### 2.7. Effect of L-Trp on Pectin Level in Flax Callus

**Figure 7:**
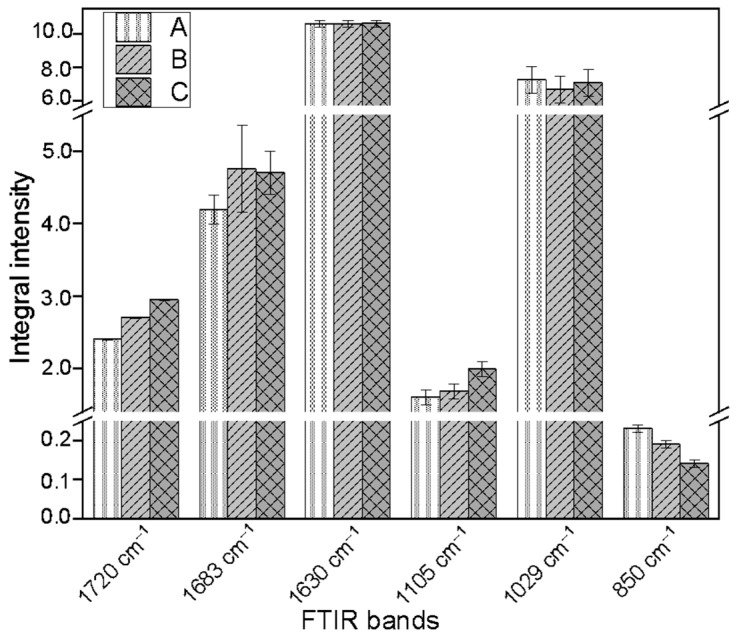
Differences in the integral intensities of FTIR absorption bands corresponding to characteristic pectin bond vibrations across the studied samples: A—control flax callus of cultivar Nike; B—callus from shaken cultures of flax with addition of 0.1 mM L-Trp; C—callus cultured in shaken cultures with addition of 1 mM L-Trp.

In the FTIR spectra, the band at 1720 cm^−1^ corresponds to stretching vibrations (ν(C=O)) of methyl-esterified carboxyl groups (Figure 7). The bands at 1683 and 1630 cm^−1^ are attributed to the asymmetric (ν_as_(COO^−^)) and symmetric (ν_s_(COO^−^)) stretching vibrations of carboxylate anions, reflecting ionized or unesterified carboxyl groups. In the fingerprint region, the band at 1105 cm^−1^ is associated with C-OH deformation vibrations (δ(C-OH)), while the band at 1029 cm^−1^ corresponds to C-O-C stretching vibrations within the glycoside linkages between galacturonic acid residues in the pectin backbone. The band at 850 cm^−1^ is typically linked to CH_2_ rocking motions (ω(CH_2_)) and out-of-plane bending (γ(CH)φ) of aromatic CH groups.

The integral intensities of the bands characteristic of pectin vibrations in the analyzed samples show only slight differences. However, it is noteworthy that the integral intensities of the band corresponding to the stretching vibrations of methyl-esterified carboxyl groups (1720 cm^−1^) are increased in the callus samples cultured with supplementation with 0.1 mM or 1 mM L-Trp, which may suggest a higher level of pectin when compared to the control (Figure 7). It should be noted that pectin ensures proper hydration of the plant cell wall [28], and thus it determines and influences the mechanical properties. The presence and composition of pectin are also important for morphogenesis and plant cell growth. It should be emphasized that auxins are phytohormones, which induce pectin methylesterification and influence properties of cell walls [36,37]. In the present study, a precursor for auxins (L-tryptophan) was added to the flax shaken cultures; therefore, the observed changes in pectin levels may be an indirect result of L-tryptophan supplementation.

## 3. Materials and Methods

### 3.1. Plant Material and Establishment of Aseptic Tissue Cultures

The research material in the present study was flax (*Linum usitatissimum* L.) of the Nike cultivar. The seeds of this plant were sterilized in 50% PPM (plant preservative mixture) and germinated in sterile conditions on MS medium [38] with addition of 1% (*w*/*v*) sucrose and solidified with 0.8% agar (the pH of the medium was adjusted to 5.8). All media were sterilized in an autoclave at 121 °C for 20 min; additionally, plant preservative mixture (750 μL/L) was added to the medium to avoid microbial contamination. The seeds were incubated in the dark in the controlled conditions of the growth chamber in 16 h day/8 h night cycles at temperatures of 21 °C (day) and 16 °C (night) in 60% humidity. Obtained seedlings were used as the starting material for callus initiation.

### 3.2. Initiation and Elicitation of Flax Callus Cultures

Obtained young seedlings (14–21 days after germination) were used for callus initiation. In this case, the flax cotyledons and hypocotyls were transferred to the solid MS medium (with 2.5% *w*/*v* sucrose and 2.5% *w*/*v* glucose) and supplemented with plant growth regulators: 0.05 mg/L naphthalene acetic acid (NAA) and 1 mg/L 6-benzylaminopurine (BAP) [39]. The cultures were incubated in the growth chamber in the conditions described above until the callus was formed. The induced callus was used for initiation of shaken cultures. Thus, calluses were transferred to liquid MS medium with 3% (*w*/*v*) sucrose, 1 mg/L BAP, 0.05 mg/L NAA and 750 μL/L PPM. The cultures were kept on orbital shakers (110 rpm, in the darkness). After 7 days, the cultures were supplemented with 0.1 mM or 1 mM L-tryptophan (Sigma-Aldrich, Saint Louis, MO, USA); no tryptophan was added to the cultures which served as controls. These two doses of L-tryptophan were analyzed because they had been tested previously in flax tissue cultures [24]. The cultivation process was terminated two weeks after the elicitor was added, because earlier observations of flax callus cultures in the applied conditions showed that the stationary phase lasts for 18–21 days [13]. The resulting callus was lyophilized and then subjected to biochemical and structural analysis.

### 3.3. Callus Proliferation Coefficient

A callus proliferation coefficient was calculated for each culture as the difference between callus fresh weight at 21 days and callus fresh weight at inoculation and expressed as percentage.

### 3.4. Determination of Photosynthetically Active Pigments in Flax Callus

The obtained calluses were collected, lyophilized and extracted with 100% acetone. The extracts were applied for measurements of absorbance, which were performed using a Tecan microplate reader (Tecan Group Ltd., Männedorf, Switzerland) at different wavelengths: 470 nm, 645 nm, and 662 nm. Finally, the calculations were performed as described by Lichtenthaler (1987) [40].

### 3.5. Determination of Amino Acid Levels in Flax Callus via HPLC-DAD Method

The amino acid content was determined using high-performance liquid chromatography with diode array detection (HPLC-DAD) following automated pre-column derivatization. Primary and secondary amino acids were derivatized using o-phthalaldehyde (OPA; 10 mg/mL in 0.4 M borate buffer with 3-mercaptopropionic acid) and 9-fluorenylmethyl chloroformate (FMOC; 2.5 mg/mL in acetonitrile), respectively, enabling their detection at 338 nm and 262 nm.

Samples were subjected to acid hydrolysis (24 h at 110 °C) in hydrochloric acid in the presence of phenol to prevent oxidative degradation of amino acids. After hydrolysis, samples were cooled to room temperature, neutralized with sodium hydroxide, filtered through 0.45 µm membrane filters, and spiked with an internal standard solution containing norvaline and sarcosine.

Chromatographic analysis was performed using an Agilent 1260 Infinity II liquid chromatograph (Agilent Technologies, Santa Clara, CA, USA) equipped with a diode array detector. Separation was achieved on a C18 column (AdvanceBio AAA, 4.6 × 100 mm, 2.7 µm LC column) maintained at 40 °C. The mobile phase consisted of (A) phosphate–borate buffer (pH 8.2) and (B) acetonitrile/methanol/water (45:45:10, *v*/*v*/*v*), applied in a gradient elution program. The flow rate was set at 1.5 mL/min, the total run time was 18 min, and the injection volume was 1 µL.

The derivatization was carried out automatically in the autosampler using sequential addition of borate buffer, sample, OPA, and FMOC reagents, followed by dilution and injection.

Detection was performed with wavelength switching between 338 nm (OPA derivatives of primary amino acids) and 262 nm (FMOC derivatives of secondary amino acids), allowing for the simultaneous determination of both groups within a single chromatographic run.

Amino acids were identified by comparing retention times with the standards. Quantification was performed using an external calibration curve in the range of 90–900 pmol/µL, with correction based on internal standards.

### 3.6. Analysis of Secondary Metabolites in Flax Callus

#### 3.6.1. Extraction of Secondary Metabolites

Ultrasound-assisted extraction (UAE) was performed to prepare extracts for analysis of polyphenol and flavonoid contents in callus cultures. Lyophilized plant tissue was extracted with 60% ethanol in an ultrasonic bath (Polsonic, Warsaw, Poland) for 45 min at 40 kHz, and the resulting extracts were used for measurements of TPC and TFC.

#### 3.6.2. Determination of Total Polyphenol Content (TPC)

The total polyphenol content (TPC) was analyzed by the Folin–Ciocâlteu method [41]. The freeze-dried calluses were extracted as described above with 60% ethanol, and the absorbance of the final sample was measured at 750 nm using a Tecan microplate reader (Tecan Group Ltd., Männedorf, Switzerland). Gallic acid (Merck, Darmstadt, Germany) served as a standard for the analysis, and measured TPC was expressed as milligrams of gallic acid equivalent (GAE eq.) per gram of plant dry weight (DW).

#### 3.6.3. Measurements of Total Flavonoid Content (TFC) in Flax Callus

In the measurements of the total flavonoid content (TFC), a method based on reaction of flavonoids with AlCl_3_ (Merck, Darmstadt, Germany) was performed, as described by Wróbel-Kwiatkowska et al. (2022) [42] and Zhishen et al. (1999) [43]. The measurements were made at 510 nm using a Tecan microplate reader. Quercetin (Merck, Darmstadt, Germany) served as a standard; thus, TFC was quantified as quercetin equivalents (QE eq.) per 100 g of plant dry weight (DW).

#### 3.6.4. Structural Analyses of Flax Callus via FTIR Spectroscopy

FTIR/ATR measurements were carried out over the spectral range of 4000–500 cm^−1^ using a Nicolet 6700 spectrometer (Thermo Fisher Scientific, Waltham, MA, USA) fitted with a portable ATR accessory. The spectra were collected at a resolution of 2 cm^−1^. Each spectrum was acquired three times and subsequently averaged. Spectral processing and analysis were performed with commercial software (OriginPro 2026, OriginLab Corp., Northampton, MA, USA). The procedure included background subtraction and deconvolution of the experimental bands into Lorentzian components. The integral intensity of the band at 2925 cm^−1^, corresponding to asymmetric stretching vibrations of methyl groups, was employed as an internal standard.

### 3.7. Statistical Analysis

Statistical analyses were performed using Student’s *t*-test (* *p* < 0.05; ** *p* < 0.01). Results are presented as the mean ± standard deviation (SD) of independent replicates.

For analysis of amino acids, expanded uncertainty was calculated at a 95% confi-dence level (k = 2). The applied method exhibited good linearity (R^2^ > 0.99), low limits of detection (LOD ≈ 0.9 pmol), and satisfactory precision (relative standard deviation, RSD < 5%). Method performance was validated by replicate analysis (n ≥ 10) and comparison with certified reference materials.

## 4. Conclusions

This study elucidated the effect of L-tryptophan on flax callus cultures in terms of biomass, accumulated bioactive compounds and structural properties of plant cell walls. Tryptophan, the largest aromatic amino acid, possesses an indole ring [44]. While it is an essential amino acid for animals, for plants, it is an important precursor of plant growth regulators, particularly auxins. L-Trp supplementation positively affected the carotenoid and total polyphenol contents, while the chlorophyll level and callus proliferation coefficients were slightly lower than for the control callus. These changes were, however, not significantly important. The results highlight the impact of tryptophan on cell wall organization and composition, suggesting that treatment with both tested concentrations of tryptophan may reduce cellulose and lignin levels but can increase pectin levels. Moreover, the findings suggested that the cell wall structure was changed, with weaker interactions between cellulose chains—possibly because tryptophan is a precursor of auxins. Finally, it can be inferred that tryptophan is an effective stimulator of polyphenol and carotenoid accumulation, compounds with high antioxidant capacity and anti-inflammation properties in flax callus cultures. These findings are important because tryptophan acts as fertilizer for plants, and moreover, they suggest that this amino acid can be used for callus shaken cultures of flax to improve the synthesis of phenolics. It should be emphasized that this study represents fundamental research in a controlled laboratory model. Future work will investigate the impact of foliar application of L-Trp on whole-plant metabolism and cell wall organization. Ultimately, these findings may help clarify the potential role of L-tryptophan in plant protection against various biotic and abiotic stresses.

## Figures and Tables

**Table 1:** The effect of L-tryptophan on amino acid level in flax callus grown in shaken cultures. The measurements were performed, as described in Section 3, using the HPLC DAD method. The results were expressed in mg/g DW.

Amino Acids (mg/g DW)	Nike − Control		Nike + 0.1 mM Trp		Nike + 1 mM Trp	
	Result	±Expanded Uncertainty	Result	±Expanded Uncertainty	Result	±Expanded Uncertainty
Aspartic acid	10.32	0.52	11.60	0.58	7.67	0.38
Glutamic acid	188.83	9.44	220.85	11.04	185.28	9.26
Serine	6.51	0.32	6.58	0.33	6.34	0.32
Histidine	0.22	0.01	0.42	0.02	0.16	0.01
Glycine	5.23	0.26	5.43	0.27	5.15	0.26
Threonine	2.44	0.12	3.09	0.15	0.9	0.04
Arginine	6.36	0.32	6.89	0.34	6.26	0.31
Alanine	7.94	0.40	8.49	0.42	8.71	0.43
Tyrosine	<0.1	-	0.17	0.01	0.13	0.01
Cysteine	3.11	0.15	3.45	0.17	3.18	0.15
Valine	5.55	0.28	5.93	0.29	5.52	0.27
Methionine	5.62	0.28	7.16	0.36	5.97	0.29
Phenylalanine	3.10	0.15	2.99	0.15	1.39	0.07
Isoleucine	1.68	0.08	1.95	0.10	0.78	0.04
Leucine	5.79	0.29	6.11	0.30	5.47	0.27
Lysine	6.84	0.34	7.02	0.35	6.61	0.33
Hydroxyproline	29.14	1.46	42.28	2.11	34.24	1.71
Proline	2.96	0.15	4.14	0.21	0.93	0.05
Tryptophan	2.14	0.11	2.73	0.14	10.28	0.51

## Data Availability

The original contributions presented in this study are included in the article. Further inquiries can be directed to the corresponding author.
